# Is intrauterine exposure to acetaminophen associated with emotional and hyperactivity problems during childhood? Findings from the 2004 Pelotas birth cohort

**DOI:** 10.1186/s12888-018-1942-1

**Published:** 2018-11-20

**Authors:** Luciana Tovo-Rodrigues, Bruna Celestino Schneider, Thais Martins-Silva, Bianca Del-Ponte, Christian Loret de Mola, Lavinia Schuler-Faccini, Fernanda Sales Luiz Vianna, Tiago N. Munhoz, Ludmila Entiauspe, Mariângela Freitas Silveira, Iná S. Santos, Alicia Matijasevich, Aluísio J. D. Barros, Luis Augusto Rohde, Andréa Dâmaso Bertoldi

**Affiliations:** 1 Postgraduate Program in Epidemiology, Universidade Federal de Pelotas, Rua Marechal Deodoro, 1160 - 3° andar, Pelotas, RS 96020-220 Brazil; 2 School of Nursing and Public Health, Universidade Federal de Pelotas, Pelotas, Brazil; 3 Hospital de Clínicas de Porto Alegre, Porto Alegre, Brazil; 4 Postgraduate Program in Genetics and Molecular Biology, Universidade Federal do Rio Grande Do Sul, Porto Alegre, Brazil; 5 Genomic Medicine Laboratory, Hospital de Clínicas de Porto Alegre, Porto Alegre, Brazil; 6 Laboratory of Research in Bioethics and Ethics in Research, Hospital de Clínicas de Porto Alegre, Porto Alegre, Brazil; 7 Department of Psychology, School of Medicine, Universidade Federal de Pelotas, Pelotas, Brazil; 8 Departamento de Medicina Preventiva, Faculdade de Medicina FMUSP, Universidade de São Paulo, São Paulo, Brazil; 9 Department of Psychiatry, Hospital de Clínicas de Porto Alegre, Universidade Federal do Rio Grande do Sul, Porto Alegre, Brazil; 10 National Institute of Developmental Psychiatry for Children and Adolescents, São Paulo, Brazil

**Keywords:** Acetaminophen (paracetamol), Prenatal exposure, Birth cohort, Behavioral symptoms

## Abstract

**Background:**

Longitudinal studies have consistently reported that prenatal exposure to acetaminophen can to lead to an increased risk of attention deficit-hyperactivity disorder during childhood. This study aimed to investigate the association between intrauterine exposure to acetaminophen and the presence of emotional and behavioral problems at the ages of 6 and 11 years in a low-middle income country.

**Methods:**

We performed a prospective longitudinal population-based study using data from the 2004 Pelotas birth cohort. From the 4231 initial cohort participants, 3722 and 3566 children were assessed at 6 and 11 years of age, respectively. The outcomes were assessed using the parent version of Strengths and Difficulties Questionnaire (SDQ). The cut-off points established for the Brazilian population were used to categorize the outcomes. Crude and adjusted odds ratio were obtained through logistic regression.

**Results:**

Acetaminophen was used by 27.5% (95% confidence interval [CI]: 26.1–28.9) of the mothers at least once during pregnancy. The prevalence of emotional problems at 6 and 11 years was 13.6 and 19.9%, respectively. For hyperactivity problems, prevalence was 13.9 and 16.1%, respectively. Intrauterine exposure to acetaminophen increased the odds of having emotional (odds ratio [OR] = 1.47; 95% CI: 1.07–2.02) and hyperactivity/inattention (OR = 1.42; 95% CI: 1.06–1.92) problems in 6-year-old boys. At the age of 11, a small decrease in the effect was observed for both outcomes after adjustment: OR = 1.31 (95% CI: 0.99–1.73) for emotional problems and OR = 1.25 (95% CI: 0.95–1.65) for hyperactivity/inattention in boys. No association for any phenotypes at both ages was observed for girls.

**Conclusion:**

The effect of intrauterine exposure to acetaminophen in emotional and hyperactivity symptoms was dependent on sex in a Brazilian cohort. While it seemed to be important for boys, mainly at 6 years of age, for girls, no association was observed.

**Electronic supplementary material:**

The online version of this article (10.1186/s12888-018-1942-1) contains supplementary material, which is available to authorized users.

## Background

Acetaminophen (or paracetamol) is one of the most commonly used analgesics worldwide [1]. It is the first choice for pain and fever medication among pregnant women [2]. In Brazil, studies reporting acetaminophen use during pregnancy are scarce [3–5]. These studies are restricted to convenience samples, and to our knowledge, there are no population-based studies that have evaluated its use.

Several birth cohort studies have reported that prenatal exposure to acetaminophen is associated with neurobehavioral and neurodevelopmental disorders during childhood [6–12]. Intrauterine exposure to acetaminophen has been associated with (i) attention-deficit/hyperactivity disorder (ADHD) [8–12]; (ii) hyperactivity/inattention and behavioral symptoms [7–11]; (iii) autism spectrum disorder (ASD) [6–10]; and (iv) impaired motor development, difficulty in communication and behavioral disorders [9–13]. In a recent meta-analysis, acetaminophen exposure during pregnancy was associated with a 20–30% increase in the risk of neurodevelopmental disorders [14]. Only one study has evaluated behavioral outcomes at two different time points during childhood [7].

Acetaminophen crosses the human placental barrier, and in vitro and in vivo findings provide additional evidence in support of observational epidemiological studies. Acetaminophen use has been shown to interfere with neurotransmitter, endocrine and immune systems, as well as with the regulation of brain-derived neurotrophic factor and cell oxidative stress, which are processes associated with brain development [15–22].

Although several variables have been included as covariates in previous studies, the results are susceptible to potential confounding effects [14], which makes it difficult to ascertain whether the use of acetaminophen is safe during pregnancy and indicates the need for more studies in the field. It is of note that current observational studies have been conducted in only European populations [23–25]. In this sense, studies in other populations, such as those from low and medium-income countries, would contribute to the knowledge in the literature and improve the current understanding of the risk of intrauterine exposure to acetaminophen.

This study aims to investigate the association between intrauterine exposure to acetaminophen and the presence of emotional and behavioral symptoms at the ages of 6 and 11 years in the 2004 Pelotas birth cohort.

## Methodology

### Target population

The present investigation is a prospective longitudinal study using data from the 2004 Pelotas birth cohort, a population-based study that documented hospital childbirths in the southern Brazilian city of Pelotas. Among the 4263 live births, 4231 were considered eligible and were included in the present study. So far, these children have been visited at the ages of 3, 12, 24, 48 and 72 months and 11 years, with follow-up rates of 95.7, 94.3, 93.5, 92.0, 90.2 and 86.6%, respectively [26, 27]. The present study used data from perinatal assessments and follow-ups at ages 6 and 11. At both follow-ups, the children underwent a wide-ranging health assessment with interviews [26].

### Use of acetaminophen during pregnancy

A standardized questionnaire was used during the perinatal evaluation conducted after the birth of the children. The mother’s use of medication during pregnancy was retrospectively framed as the following question: “*Did you use any medications during pregnancy?*” For positive answers, the mothers were asked to report the names of all medicines used during pregnancy, as well as the beginning and end of use. Our exposure variable was defined as medication composed of acetaminophen that was used at least once during pregnancy.

### Outcome measurements

Behavioral symptoms were evaluated using the standardized scores from the Strengths and Difficulties Questionnaire (SDQ). The SDQ is a screening questionnaire, which measures 25 psychological attributes divided into 5 scales: inattention/hyperactive symptoms, conduct problems, emotional symptoms, peer relationship problems, and prosocial behavior. All attributes except prosocial behavior are summed to create a total difficulties score. The questionnaire was adapted and previously validated for the Brazilian population of children and teenagers aged between 4 and 16 years [28].

Trained psychologists administered the SDQ in a standardized manner to the parents or caregivers during each follow-up. The cut-off points established for the Brazilian population were used to categorize the general difficulties scale and the subscales as follows: ≥17 for the total difficulties score, ≥5 for the emotional problems score, ≥4 for the conduct problems score, ≥7 for the inattention/hyperactivity score, ≥4 for the peer problems score, and ≤ 4 for the prosocial score.

### Confounding factors

The analyses included variables associated with psychiatric disorders and differential use of acetaminophen as follows: National Economic Index score (an economic indicator extensively used in Brazil based on information from the 2000 demographic census by the Brazilian Institute for Geography and Statistics) [29]; sex (male, female); mother’s educational level (years of school attendance); mother’s age (years); mother’s skin color (white, black or other); mother’s parity (number of children), smoking activity during pregnancy (“Did you smoke during pregnancy?”; yes or no); alcohol consumption during pregnancy (“Did you drink alcoholic beverages during pregnancy?”; yes or no); mood issues during pregnancy (“During pregnancy, did you have depression or suffer from anxiety?”; “no”, “yes, not treated” or “yes, treated” and categorized into yes or no responses); infections during pregnancy (yes or no); prepregnancy body mass index (BMI); and other nonsteroidal analgesic use during pregnancy (yes or no).

### Statistical analyses

Statistical analyses were performed using Stata software (version 12.1). The analyses included children who had information available regarding maternal use of acetaminophen during pregnancy and who were assessed using the SDQ at the ages of 6 and 11 years (*N* = 3470 and *N* = 3447, respectively). The crude and adjusted analyses between exposure to acetaminophen and the outcomes were performed using logistic regression. Specific effects of acetaminophen in boys and girls were also tested.

As a sensitivity analysis, to test the specificity of acetaminophen compared to other nonsteroidal analgesics, a variable regarding the use of other analgesics was created and used as a comparative exposure. All models using intrauterine acetaminophen exposure were also adjusted for this variable. Moreover, to assess the extent of the imprecision in the mood problem information (collected perinatally), we used information from the Edinburgh Postnatal Depression Scale (EPDS) [30], which has been validated for Brazilian populations [31], as a sensitivity analysis. Data were gathered when the child was 12 months old, as this time point it correlates highly with the mood problem information collected at the perinatal interview. The cut-off of 10 points, which is the recommended cut-off for screening in the Brazilian population [31], was used. The EPDS information was added as a covariable in the models, thus replacing the variable that was gathered perinatally.

## Results

Acetaminophen was used during pregnancy at a frequency of 27.5% (95% confidence interval [CI]: 26.1–28.9). The prevalence of behavioral disorders assessed through the total difficulties scale of the SDQ was 10.8% (95% CI: 9.7–11.8) at the age of 6 and 13.9% (95% CI: 12.8–15.1) at the age of 11 (Table 1). The sociodemographic, behavioral, and maternal health characteristics are also shown in Table 1. The associations between the covariables and both the exposure and the main outcomes of the study are shown in Additional file 1: Table S1.

**Table 1:** Sociodemographic characteristics, maternal health, and use of acetaminophen and other analgesics during pregnancy among mothers of boys and girls in the 2004 Pelotas birth cohort who were assessed by SDQ at the follow-ups at 6 and 11 years of age

Maternal characteristics	Follow-up at the age of 6 years		Follow-up at the age of 11 years	
	Boys (*N* = 1812) N (%)	Girls (*N* = 1658) N (%)	Boys (*N* = 1781) N (%)	Girls (*N* = 1666) N (%)
Age (years)				
≤ 24	818 (45.14)	761 (45.93)	793 (44.53)	750 (45.07)
25–29	416 (22.96)	370 (22.33)	402 (22.57)	374 (22.48)
30–34	388 (21.41)	343 (20.70)	393 (22.07)	355 (21.33)
≥ 35	190 (10.49)	183 (11.04)	193 (10.84)	185 (11.12)
Educational level (years)				
0–4	268 (14.91)	250 (15.26)	257 (14.54)	248 (15.07)
5–8	753 (41.88)	697 (42.55)	726 (41.09)	692 (42.04)
9–11	589 (32.76)	546 (33.33)	599 (33.90)	560 (34.02)
≥ 12	188 (10.46)	145 (8.85)	185 (10.47)	146 (8.87)
National Economic Index				
1 (higher)	341 (18.82)	311 (18.76)	325 (18.25)	302 (18.13)
2	362 (19.98)	366 (22.07)	355 (19.93)	363 (21.79)
3	357(19.70)	342 (20.63)	348 (19.54)	349 (20.95)
4	389 (21.47)	329 (19.84)	386 (21.67)	337 (20.23)
5 (lower)	363 (20.03)	310 (19.70)	367 (20.61)	315 (18.91)
Skin color				
White	1125 (62.92)	1016 (61.80)	1106 (62.98)	1015 (61.52)
Black	298 (16.67)	276 (16.79)	291 (16.57)	275 (16.67)
Other^a^	365 (20.41)	352 (21.41)	359 (20.44)	360 (21.82)
Smoking during pregnancy				
No	1332 (73.51)	1202 (72.50)	1320 (74.12)	1212 (72.75)
Yes	480 (26.49)	456 (27.50)	461 (25.88)	454 (27.25)
Use of alcohol during pregnancy				
No	1750 (96.58)	1605 (96.80)	1720 (96.57)	1616 (97.00)
Yes	62 (3.42)	53 (3.20)	61 (3.43)	50 (3.00)
Parity				
1	751 (41.45)	623 (37.60)	740 (41.55)	618 (37.12)
2	477 (26.32)	443 (26.74)	472 (26.50)	454 (27.27)
3 or more	584 (32.23)	591 (35.67)	569 (31.95)	593 (35.62)
Pre-gestational BMI				
Underweight	75 (4.49)	69 (4.54)	75 (4.59)	66 (4.32)
Normal weight	1025 (61.34)	909 (59.76)	996 (60.95)	914 (59.82)
Overweight	402 (24.06)	350 (23.01)	401 (24.54)	358 (23.43)
Obese	169 (10.11)	193 (12.69)	162 (9.91)	190 (12.43)
Mood symptoms during pregnancy				
No	1362 (75.17)	1254 (75.72)	1351 (75.86)	1268 (76.20)
Yes	450 (24.83)	402 (24.28)	430 (24.14)	396 (23.80)
Infections during pregnancy				
No	1066 (58.96)	974 (58.89)	1061 (59.71)	975 (58.63)
Yes	742 (41.04)	680 (41.11)	716 (40.29)	688 (41.37)
Use of acetaminophen during pregnancy				
Never	1287 (71.03)	1218 (73.46)	1259 (70.69)	1229 (73.77)
At least once	525 (28.97)	440 (26.54)	522 (29.31)	437 (26.23)
Use of other analgesics during pregnancy				
Never	1430 (79.53)	1310 (79.25)	1402 (79.21)	1311 (78.83)
At least once	368 (20.47)	343 (20.75)	368 (20.79)	352 (21.17)
Outcomes				
Total SDQ (score ≥ 17 pts)	212 (11.70)	161 (9.71)	288 (16.17)	192 (11.52)
Emotional symptoms (score ≥ 5 pts)	237 (13.08)	235 (14.17)	339 (19.03)	347 (20.83)
Conduct Problems (score ≥ 4 pts)	295 (16.28)	220 (13.27)	253 (14.21)	198 (11.88)
Hyperactivity/inattention (score ≥ 7 pts)	288 (15.89)	193 (11.64)	367 (20.61)	189 (11.34)
Peer relationship problems (score pts)	277 (15.29)	213 (12.85)	269 (15.10)	190 (11.40)
Prosocial behavior (score ≤ 4 pts)	23 (1.27)	18 (1.09)	28 (1.57)	20 (1.20)

^a^Other skin color: self-classification as brown, yellow or indigenous

Crude and adjusted analyses for the total sample are presented in Table 2. No associations between acetaminophen exposure and total problems or SDQ subscales were observed after adjustment. However, a significant interaction effect was observed for the emotional (*p* = 0.046) and hyperactivity/inattention (*p* = 0.018) subscales at 6 years old. Further analyses were conducted and stratified according to the sex of the children.

**Table 2:** Association between use of acetaminophen by the mother during pregnancy and mental health outcomes at the age of 6 years (*N* = 3470)

Outcomes	Frequency of outcome according to use of acetaminophen during pregnancy N (%)		Crude analysis	Adjusted analysis^a^		*p*-value for sex – use of acetaminophen interaction term^b^
	Exposed n (%)	Not exposed n (%)	OR (95% CI)	OR (95% CI)	*p*	
6 years old						
Total SDQ (score ≥ 17 pts)	102 (10.57)	271 (10.82)	0.97 (0.77–1.24)	1.15 (0.88–1.50)	0.313	0.454
Emotional symptoms (score ≥ 5 pts)	137 (14.20)	335 (13.37)	1.07 (0.87–1.33)	1.15 (0.91–1.46)	0.232	**0.046**
Conduct problems (score ≥ 4 pts)	119 (12.33)	396 (15.81)	0.75 (0.60–0.93)	0.86 (0.68–1.10)	0.222	0.572
Hyperactivity/inattention (score ≥ 7 pts)	137 (14.20)	344 (13.73)	1.04 (0.84–1.29)	1.10 (0.87–1.39)	0.427	**0.018**
Peer relationship problems (score ≥ 4 pts)	124 (12.85)	366 (14.61)	0.86 (0.69–1.07)	1.08 (0.85–1.38)	0.517	0.140
Prosocial behavior (score ≤ 4 pts)	8 (0.83)	33 (1.32)	0.63 (0.29–1.36)	0.63 (0.27–1.49)	0.376	0.967
11 years old						
Total SDQ (score ≥ 17 pts)	138 (14.39)	342 (13.75)	1.06 (0.85–1.30)	1.19 (0.94–1.50)	0.157	0.811
Emotional symptoms (score ≥ 5 pts)	213 (22.21)	473 (19.01)	1.22 (1.01–1.46)	1.20 (0.98–1.46)	0.078	0.313
Conduct problems (score ≥ 4 pts)	108 (11.26)	343 (13.79)	0.79 (0.63–1.00)	0.93 (0.72–1.20)	0.561	0.264
Hyperactivity/inattention (score ≥ 7 pts)	165 (17.21)	391 (15.72)	1.12 (0.91–1.36)	1.20 (0.96–1.49)	0.112	0.797
Peer relationship problems (score ≥ 4 pts)	131 (13.66)	328 (13.18)	1.04 (0.84–1.30)	1.21 (0.95–1.53)	0.123	0.781
Prosocial behavior (score ≤ 4 pts)	8 (0.83)	40 (1.61)	0.52 (0.24–1.10)	0.79 (0.36–1.75)	0.565	0.151

^a^Logistic regression adjusted according to sex, maternal age, parity, national economic index, maternal educational level, smoking during pregnancy, alcohol consumption during pregnancy, maternal skin color, infection during pregnancy, pre-gestational BMI, presence of maternal mood issue and use of other analgesics during pregnancy. N after adjustment: 3103. ^b^*p*-values marked in bold denote statistically significant results

Table 3 presents the association between prenatal exposure to acetaminophen and the outcomes evaluated in 6-year-old boys and girls. At this age, for boys, the use of acetaminophen during pregnancy was associated with emotional (OR = 1.47; 95% CI: 1.07–2.02), as well as with hyperactivity/inattention (OR = 1.42; 95% CI: 1.06–1.92), problems in the adjusted model. The use of acetaminophen during pregnancy was not associated with other outcomes. For girls, no association was observed.

**Table 3:** Association between use of acetaminophen by mother during pregnancy and mental health outcomes at the age of 6 years among boys (*N* = 1812) and girls (*N* = 1658)

Outcomes	Frequency of outcome according to use of acetaminophen during pregnancy N (%)		Crude analysis	Adjusted analysis^a^	
	Exposed n (%)	Not exposed n (%)	OR (95% CI)	OR (95% CI)	*p* ^b^
Boys					
Total SDQ (score ≥ 17 pts)	63 (12.00)	149 (11.58)	1.04 (0.76–1.43)	1.28 (0.90–1.83)	0.163
Emotional symptoms (score ≥ 5 pts)	83 (15.81)	154 (11.97)	1.38 (1.04–1.84)	1.47 (1.07–2.02)	**0.018**
Conduct problems (score ≥ 4 pts)	72 (13.71)	223 (17.33)	0.76 (0.57–1.02)	0.93 (0.68–1.27)	0.641
Hyperactivity/inattention (score ≥ 7 pts)	93 (17.71)	195 (15.15)	1.21 (0.92–1.58)	1.42 (1.06–1.92)	**0.020**
Peer relationship problems (score ≥ 4 pts)	81 (15.43)	196 (15.23)	1.02 (0.77–1.35)	1.25 (0.91–1.71)	0.167
Prosocial behavior (score ≤ 4 pts)	5 (0.95)	18 (1.40)	0.68 (0.25–1.84)	0.60 (0.19–1.88)	0.376
Girls					
Total SDQ (score ≥ 17 pts)	39 (8.86)	122 (10.02)	0.87 (0.60–1.28)	1.02 (0.67–1.54)	0.943
Emotional symptoms (score ≥ 5 pts)	54 (12.27)	181 (14.86)	0.80 (0.58–1.11)	0.90 (0.63–1.28)	0.557
Conduct problems (score ≥ 4 pts)	47 (10.68)	173 (14.20)	0.72 (0.51–1.02)	0.79 (0.54–1.16)	0.226
Hyperactivity/inattention (score ≥ 7 pts)	44 (10.00)	149 (12.23)	0.80 (0.56–1.14)	0.76 (0.51–1.12)	0.165
Peer relationship problems (score ≥ 4 pts)	43 (9.77)	170 (13.96)	0.67 (0.47–0.95)	0.88 (0.59–1.31)	0.535
Prosocial behavior (score ≤ 4 pts)	3 (0.68)	15 (1.23)	0.55 (0.16–1.91)	0.73 (0.20–2.69)	0.630

^a^Logistic regression adjusted according to maternal age, parity, national economic index, maternal educational level, smoking during pregnancy, alcohol during pregnancy, maternal skin color, infection during pregnancy, pre-gestational BMI, presence of maternal mood disorder and use of other analgesics during pregnancy. N after adjustment: 1619 for boys; 1484 for girls. ^b^*p*-values marked in bold denote statistically significant results

The effect sizes in the assessment at 11 years of age were slightly smaller than those of the 6 year-old assessment, and most of the associations that were observed at 6 years did not reach significance at 11 years (Table 4). Among boys, the crude analysis showed an effect for emotional problems (OR = 1.33; 95% CI: 1.03–1.71). However, this effect lost significance after adjustment (OR = 1.31; 95% CI: 0.99–1.73). Regarding the hyperactivity/inattention subscale, the estimates for crude and adjusted models did not reach statistical significance. Acetaminophen use during pregnancy was not associated with other outcomes for either boys or girls in the adjusted model.

**Table 4:** Association between use of acetaminophen by mother during pregnancy and mental health outcomes at the age of 11 years among boys (*N* = 1781) and girls (*N* =  1666)

Outcomes	Frequency of outcome according to use of acetaminophen during pregnancy N (%)		Crude analysis	Adjusted analysis^a^	
	Exposed n (%)	Not exposed n (%)	OR (95% CI)	OR (95% CI)	*p*
Boys					
Total SDQ (score ≥ 17 pts)	89 (17.05)	199 (15.81)	1.09 (0.83–1.44)	1.21 (0.89–1.64)	0.228
Emotional symptoms (score ≥ 5 pts)	116 (22.22)	223 (17.71)	1.33 (1.03–1.71)	1.31 (0.99–1.73)	0.052
Conduct problems (score ≥ 4 pts)	68 (13.03)	185 (14.69)	0.87 (0.64–1.17)	1.08 (0.77–1.51)	0.664
Hyperactivity/inattention (score ≥ 7 pts)	115 (22.03)	252 (20.02)	1.13 (0.88–1.45)	1.25 (0.95–1.65)	0.115
Peer relationship problems (score ≥ 4 pts)	78 (14.94)	191 (15.17)	0.98 (0.74–1.31)	1.16 (0.85–1.59)	0.339
Prosocial behavior (score ≤ 4 pts)	3 (0.57)	25 (1.99)	0.29 (0.86–0.95)	0.47 (0.14–1.63)	0.234
Girls					
Total SDQ (score ≥ 17 pts)	49 (11.21)	143 (11.64)	0.95 (0.68–1.35)	1.16 (0.80–1.69)	0.426
Emotional symptoms (score ≥ 5 pts)	97 (22.20)	250 (20.34)	1.12 (0.85–1.46)	1.08 (0.81–1.43)	0.624
Conduct problems (score ≥ 4 pts)	40 (9.15)	158 (12.86)	0.68 (0.47–0.98)	0.77 (0.52–1.14)	0.192
Hyperactivity/inattention (score ≥ 7 pts)	50 (11.44)	139 (11.31)	1.01 (0.72–1.43)	1.14 (0.79–1.64)	0.498
Peer relationship problems (score ≥ 4 pts)	53 (12.13)	137 (11.15)	1.10 (0.78–1.54)	1.30 (0.90–1.89)	0.164
Prosocial behavior (score ≤ 4 pts)	5 (1.14)	15 (1.22)	0.94 (0.34–2.59)	1.38 (0.47–4.07)	0.562

^a^Logistic regression adjusted according to maternal age, parity, national economic index, maternal educational level, smoking during pregnancy, alcohol comsumption during pregnancy, maternal skin color, infection during pregnancy, pre-gestational BMI, presence of maternal mood issue and use of other analgesics during pregnancy. N after adjustment: 1585 boys and 1494 girls

### Sensitivity analyses

To test the specificity of the effect of acetaminophen exposure during intrauterine life on the outcomes of emotional symptoms and hyperactivity, we performed another analysis to test the effect of other nonsteroidal analgesics on the same outcomes. No associations between nonsteroidal analgesics and emotional symptoms (OR_adjusted_ = 1.09; 95% CI: 0.77–1.55) or hyperactivity symptoms (OR_adjusted_ = 1.07; 95% CI: 0.77–1.48) were found. No association was observed for either sex for any of the other outcomes (results not shown). In addition, nonsteroidal analgesic use was not associated with any outcomes at 11 years of age.

The second sensitivity analysis aimed to test the imprecision of reported mood problems during pregnancy that were collected perinatally. We performed this analysis by replacing the mood problems variable during pregnancy with the maternal depression information assessed with EPDS in the statistical models. Adjusted analysis results are presented in Table 5. Analyses did not change substantially. The same associations as before were observed in the 6 year-old assessment. In the 11-year-old assessment, the magnitude of the effect of acetaminophen on emotional problems in boys was greater than that in the previous analysis, and the adjusted analysis became significant (OR = 1.33; 95% CI: 1.01–1.76).

**Table 5:** Sensitivity analysis considering Edinburgh Postnatal Depression Scale at 12 months follow-up interview for the association between exposure to acetaminophen during pregnancy and SDQ total and subscales problems of children at 6 and 11 years of age

Outcomes	6 years		11 years	
	Adjusted analysis^a^		Adjusted analysis^a^	
	OR (95% CI)	*P* ^b^	OR (95% CI)	*P* ^b^
Boys				
Total SDQ (score ≥ 17 pts)	1.22 (0.85–1.75)	0.291	1.22 (0.89–1.66)	0.221
Emotional symptoms (score ≥ 5 pts)	1.42 (1.02–1.97)	**0.038**	1.33 (1.01–1.76)	**0.045**
Conduct problems (score ≥ 4 pts)	0.89 (0.64–1.24)	0.503	1.09 (0.78–1.54)	0.609
Hyperactivity/inattention (score ≥ 7 pts)	1.43 (1.05–1.94)	**0.022**	1.25 (0.94–1.65)	0.121
Peer relationship problems (score ≥ 4 pts)	1.21 (0.88–1.67)	0.251	1.14 (0.83–1.57)	0.415
Prosocial behavior (score ≤ 4 pts)	0.65 (0.21–2.03)	0.457	0.48 (0.14–1.66)	0.246
Girls				
Total SDQ (score ≥ 17 pts)	0.99 (0.65–1.51)	0.954	1.14 (0.78–1.67)	0.503
Emotional symptoms (score ≥ 5 pts)	0.90 (0.63–1.28)	0.554	1.06 (0.79–1.42)	0.694
Conduct problems (score ≥ 4 pts)	0.81 (0.55–1.19)	0.290	0.73 (0.49–1.10)	0.135
Hyperactivity/inattention (score ≥ 7 pts)	0.76 (0.51–1.13)	0.175	1.10 (0.76–1.61)	0.613
Peer relationship problems (score ≥ 4 pts)	0.89 (0.60–1.32)	0.559	1.31 (0.90–1.91)	0.155
Prosocial behavior (scor e ≤ 4 pts)	0.75 (0.20–2.80)	0.663	1.39 (0.47–4.15)	0.553

^a^Logistic regression adjusted according to maternal age, parity, national economic index, maternal educational level, smoking during pregnancy, alcohol consumption during pregnancy, maternal skin color, infection during pregnancy, pre-gestational BMI, presence of maternal depression and use of other analgesics during pregnancy. N after adjustment: 1.555 for boys and 1.137 for girls at 6 years; 1.523 for boys and 1.442 for girls at 11 years. ^b^*p*-values marked in bold denote statistically significant results

## Discussion

In this study, we examined the association between acetaminophen exposure during pregnancy and the behavioral outcomes of offspring in a Brazilian birth cohort. A differential effect for sex and exposure to acetaminophen was observed. In 6-year-old boys, intrauterine exposure to acetaminophen led to increased odds of having emotional and hyperactivity/inattention problems. At the age of 11, a slight reduction in the effect size was observed. For girls, the results were nonsignificant at both follow-up times for all the considered outcomes. These findings suggest that acetaminophen exposure in the uterus may play a role in behavioral disorders in childhood, mainly in boys.

Acetaminophen is one of the most prescribed and used medications for pregnant women in Brazil [32, 33]. Comparing our results with those of previous studies, an important difference is in exposure prevalence. Although over 50% of pregnant women report using acetaminophen in the United States and Denmark [8, 34–36], we found that 27.5% of Brazilian mothers used this substance during pregnancy. We could not find Brazilian population-based studies reporting acetaminophen use during pregnancy.

Evidence from different prospective cohort studies has shown an association between acetaminophen use during pregnancy and neurobehavioral outcomes in childhood [6–12]. Three studies evaluated behavioral symptoms using the SDQ. In 7-year-old children, Liew et al. [8] observed an association between prenatal exposure to acetaminophen and higher total difficulties, conduct problems, and hyperactivity. The authors also observed increased risk when the medication was used over more than one trimester, especially near the end of the pregnancy. In addition, they found a correlation between increased frequency of use and increased risk. Stergiakouli et al. [11] demonstrated that children exposed to acetaminophen during the second and third trimesters were at higher risk of multiple behavioral difficulties, including hyperactivity and conduct problems at 7 years of age. Prenatal exposure to acetaminophen during a 32-week pregnancy was also associated with emotional symptoms. Using parent-reported SDQ scores, Thompson et al. [7] observed that acetaminophen was a risk factor for total difficulties, emotional symptoms, and conduct problems at 7 years. At age 11, however, only an association with the parent-reported emotional scale was observed.

Animal studies have supported epidemiological observational studies, providing biological plausibility for the findings. Acetaminophen is known to freely cross the placenta [37]. Recently, exposure to acetaminophen during intrauterine life was reported to affect the modulation of neurotransmission in rats [17]. Effects on neurodevelopment and behavior have also been observed in adult rats exposed to acetaminophen during intrauterine life [15, 16]. Defects in cognitive function and deficient levels of brain-derived neurotrophic factor (BDNF) were observed in rats that were administered acetaminophen during neonatal life [38]. Interference in the regulation of cellular oxidative stress and the endocrine and immune systems by acetaminophen has also been suggested as a mechanism that could lead to neurodevelopment disorders [18–22, 39].

Sex-related differences in symptomatology have been reported for both ADHD and emotional problems. ADHD is more commonly diagnosed in boys than in girls [40], and girls with ADHD are more likely than boys to present inattentive ADHD and separation anxiety disorder. Boys present higher rates of hyperactivity and comorbidity for disorders such as oppositional defiant disorder and conduct disorder [40, 41]. Emotional problems in early adolescence, such as anxiety and depression, are more frequent in girls than in boys [42–45]. In this study, we tested whether sex could modify the effect of acetaminophen on behavioral problems. An interaction between acetaminophen exposure and sex was observed for the hyperactivity and emotional scales, suggesting that acetaminophen may have different effects on these problems in boys and girls. Stratified analyses suggested an effect of acetaminophen on the hyperactivity and emotional scales only in boys.

A similar effect was reported for ASD symptoms in a Spanish cohort [10]. The findings in Spain revealed that exposed boys had more ASD symptoms compared to exposed girls, who showed decreased scores. Differential acetaminophen metabolization between males and females has already been reported and may play a role in this difference, as suggested by Avella-Garcia et al. [10]. Studies on animals have demonstrated that male mice show higher toxicity levels than females after acetaminophen administration [46]. Other sex differences rely on a putatively increased vulnerability to stressors [47] and to the effect of acetaminophen on testosterone regulation [39, 48], neurodevelopment, and brain masculinization [15] in male brains at the beginning of life. Thus, the hypothesis that endocrine disruption influences testicular function and the production of androgens, which further affects brain development in males, could be a plausible mechanism for the present findings.

The results presented in this study suggest that the magnitude of the effect on emotional and hyperactivity/inattention problems slightly decreased from age 6 to 11. Our sensitivity analysis using EPDS showed that the absence of an association in the adjusted analysis at the age of 11 may be due to a lack of power in the analyses for emotional problems. Our results at different ages are in line with the results reported by Thompson et al. [7], which showed differences between both ages for the parent-reported symptoms. Studies evaluating other age groups, including adolescence, are necessary to better understand the relationship between the exposure and the evaluated outcomes.

The results should be interpreted considering certain limitations. The first limitation relates to the retrospective gathering of data on medication use during pregnancy, which may have resulted in difficulty recalling acetaminophen use. Thus, the low prevalence of acetaminophen use observed in this study may be a result of underreporting. We compared our data with that collected in the same population, in 2015, by another birth cohort study (2015 Pelotas Birth Cohort). In this birth cohort, acetaminophen use information was assessed prospectively during pregnancy, as well as in the perinatal interview (retrospectively). The prevalence for use in the perinatal interview was 51% (unpublished data), which agrees with most of the data from published studies [8, 32–34]. Considering that underreporting use of acetaminophen may be a limitation for the exposure definition in our study, we expect that effect size observed in the association analyses is underestimated. Therefore, false negative results are more likely to be observed than false positives regarding this limitation. Second, both the dose-response and timing of exposure associations (higher risks for the third trimester of pregnancy exposure) had been reported previously [8–11, 13]. However, because of possible memory issues, our data did not allow for the evaluation of exposure according to the trimester of pregnancy.

Furthermore, we cannot rule out the effect of using other medications during pregnancy on behavioral outcomes. However, other types of nonsteroidal analgesics were not associated with worse outcomes. Nonsteroidal analgesic use was also included as a covariable in the models. Thus, it is unlikely that co-medication with other analgesics influenced the findings. These results are consistent with the specificity of acetaminophen observed by Thompson et al. [7] and with the results from previous studies that used the same adjustment approach with other analgesics [8]. It has also been shown that children whose mothers have externalizing disorders (e.g., conduct disorder or drug/alcohol dependence) are at an increased risk for developing behavior problems [49]. Although we do not have information regarding externalizing problems in mothers, we included smoking and drinking behavior during pregnancy, which could be considered as proxies. Other psychological variables from mothers would also improve the robustness of our findings. Fever and inflammation during pregnancy are known risk factors for the development of neurological diseases [50]. Werenberg-Dreier et al. [51] showed that exposure to fever and infection over specific timeframes during pregnancy was associated with the occurrence of ADHD. Although the women in the present study reported using the medication, their reasons for doing so were not available. This information would also be important to improve the robustness of our findings. However, this information was not collected in 2004.

Another critical limitation, which has been greatly discussed in the literature, was the presence of residual confounding due to socioeconomic variables. Additional file 1: Table S1 shows socioeconomic variables, including educational level, economic index, skin color, and parity, which are variables that behave as proxies for socioeconomic position (SEP) in Brazil. These variables were all associated with the use of acetaminophen during pregnancy and with most of the outcomes at different ages, especially hyperactivity/inattention problems. While other variables were not statistically associated with our main exposure, we included some of them in the adjusted models given recent evidence suggesting them as possible confounders of this association. However, these variables might not be as important as SEP variables in our models. It is unlikely that residual confounding due to a lack of control over other SEP measurements, such as income and work, may have biased our results. Models were adjusted for variables associated with exposure. Further, the results considered most of the effects of SEP. In addition, crude and adjusted measures of the association between acetaminophen exposure and emotional problems at 11 years of age are very similar, reinforcing the idea that the association was, probably, not confounded by these variables.

## Conclusions

The use of acetaminophen in pregnant mothers was associated with subsequent emotional and hyperactivity symptoms in their 6-year-old boys. A suggestive association was observed in 11-year-old boys. Further studies that gather more precise information concerning dose and time of exposure among pregnant women are necessary to establish the long-term effects of maternal acetaminophen use during pregnancy on offspring.

## Additional file

Additional file 1: **Table S1.** Association analysis between covariates, exposure and relevant outcomes. (DOCX 22 kb)
