# Supplementary material for: Is intrauterine exposure to acetaminophen associated with emotional and hyperactivity problems during childhood? Findings from the 2004 Pelotas birth cohort
Source: BMC Psychiatry. 2018 Nov 20;18:368. doi: 10.1186/s12888-018-1942-1 (PMC6245767; doi:10.1186/s12888-018-1942-1)
Supplement: Supplementary file 1 — Table S1. Association analysis between covariates, exposure and relevant outcomes. (DOCX 22 kb) [file 12888_2018_1942_MOESM1_ESM.docx]

Additional file 1: **Table S1** -Univariate association between covariates and exposure and relevant outcomes

|  | Acetaminophen use during pregnancy | | SDQ Emotional symptoms subscale (score ≥ 5 pts)  (at 6 years of age) | | SDQ Emotional symptoms (score ≥ 5 pts) subscale  (at 11 years of age) | | SDQ Hyperactivity/inattention (score ≥ 7 pts) subscale  (at 6 years of age) | | SDQ Hyperactivity/inattention (score ≥ 7 pts) subscale  (at 11 years of age) | |
| --- | --- | --- | --- | --- | --- | --- | --- | --- | --- | --- |
|  | No | Yes | No | Yes | No | Yes | No | Yes | No | Yes |
|  | N (%) | N (%) | N (%) | N (%) | N (%) | N (%) | N (%) | N (%) | N (%) | N (%) |
| 3 quantiles of schooling | P<0.001 | | P=0.002 | | P=0.007 | | P<0.001 | | P<0.001 | |
| 1 | 573 (37.9) | 147 (24.3) | 524 (32.6) | 105 (43.8) | 456 (30.9) | 135 (38.9) | 507 (32.7) | 122 (41.4) | 440 (30.6) | 151 (39.5) |
| 2 | 479 (31.7) | 211 (34.8) | 542 (33.7) | 73 (30.4) | 523 (35.5) | 98 (28.2) | 507 (32.7) | 108 (36.6) | 482 (33.5) | 139 (36.4) |
| 3 | 458 (30.3) | 248 (40.9) | 541 (33.7) | 62 (25.8) | 495 (33.6) | 114 (32.9) | 538 (34.7) | 65 (22) | 517 (35.9) | 92 (24.1) |
| 3 quantiles of national economic index | P<0.001 | | P<0.001 | | P=0.504 | | P<0.001 | | P<0.001 | |
| 1 | 553 (36.3) | 139 (22.7) | 499 (30.8) | 91 (37.8) | 453 (30.4) | 114 (32.9) | 473 (30.2) | 117 (39.7) | 416 (28.7) | 151 (39.4) |
| 2 | 486 (31.9) | 218 (35.7) | 535 (33) | 97 (40.2) | 508 (34.1) | 121 (34.9) | 517 (33) | 115 (39) | 490 (33.7) | 139 (36.3) |
| 3 | 483 (31.7) | 254 (41.6) | 586 (36.2) | 53 (22) | 527 (35.4) | 112 (32.3) | 576 (36.8) | 63 (21.4) | 546 (37.6) | 93 (24.3) |
| Parity (number of pregnancies) | P<0.001 | | P=0.313 | | P=0.693 | | P<0.001 | | P=0.003 | |
| 1 | 604 (39.7) | 279 (45.7) | 665 (41) | 111 (46.1) | 615 (41.3) | 151 (43.5) | 630 (40.2) | 146 (49.5) | 607 (41.8) | 159 (41.5) |
| 2-3 | 620 (40.7) | 263 (43) | 685 (42.3) | 91 (37.8) | 628 (42.2) | 138 (39.8) | 685 (43.7) | 91 (30.8) | 626 (43.1) | 140 (36.6) |
| 4 or more | 298 (19.6) | 69 (11.3) | 270 (16.7) | 39 (16.2) | 245 (16.5) | 58 (16.7) | 251 (16) | 58 (19.7) | 219 (15.1) | 84 (21.9) |
| Skin colour | P=0.002 | | P=0.020 | | P=0.110 | | P=0.011 | | P=0.003 | |
| White | 902 (60) | 411 (68.2) | 1026 (64.1) | 131 (55.7) | 936 (63.8) | 205 (59.9) | 997 (64.4) | 160 (55.6) | 930 (64.9) | 211 (56.1) |
| Black | 271 (18) | 82 (13.6) | 264 (16.5) | 41 (17.4) | 246 (16.8) | 53 (15.5) | 243 (15.7) | 62 (21.5) | 218 (15.2) | 81 (21.5) |
| Other | 330 (22) | 110 (18.2) | 311 (19.4) | 63 (26.8) | 286 (19.5) | 84 (24.6) | 308 (19.9) | 66 (22.9) | 286 (19.9) | 84 (22.3) |
| Alcohol intake during pregnancy | P=0.474 | | P=0.692 | | P=0.069 | | P=0.518 | | P=0.467 | |
| No | 1465 (96.3) | 592 (96.9) | 1565 (96.6) | 234 (97.1) | 1444 (97) | 330 (95.1) | 1512 (96.6) | 287 (97.3) | 1406 (96.8) | 368 (96.1) |
| Yes | 57 (3.7) | 19 (3.1) | 55 (3.4) | 7 (2.9) | 44 (3) | 17 (4.9) | 54 (3.4) | 8 (2.7) | 46 (3.2) | 15 (3.9) |
| Smoking during pregnancy | P=0.165 | | P=0.022 | | P=0.314 | | P=0.002 | | P<0.001 | |
| No | 1101 (72.3) | 460 (75.3) | 1202 (74.2) | 162 (67.2) | 1107 (74.4) | 249 (71.8) | 1169 (74.6) | 195 (66.1) | 1107 (76.2) | 249 (65) |
| Yes | 421 (27.7) | 151 (24.7) | 418 (25.8) | 79 (32.8) | 381 (25.6) | 98 (28.2) | 397 (25.4) | 100 (33.9) | 345 (23.8) | 134 (35) |
| Mood symptoms during pregnancy | P=0.243 | | P<0.001 | | P<0.001 | | P=0.007 | | P<0.001 | |
| No | 1148 (75.4) | 446 (73) | 1241 (76.6) | 155 (64.3) | 1163 (78.2) | 227 (65.4) | 1193 (76.2) | 203 (68.8) | 1130 (77.8) | 260 (67.9) |
| Yes | 374 (24.6) | 165 (27) | 379 (23.4) | 86 (35.7) | 325 (21.8) | 120 (34.6) | 373 (23.8) | 92 (31.2) | 322 (22.2) | 123 (32.1) |
| Infection during pregnancy | P=0.138 | | P=0.003 | | P=0.082 | | P=0.166 | | P=0.038 | |
| No | 911 (60.1) | 345 (56.6) | 972 (60.2) | 121 (50.2) | 899 (60.6) | 192 (55.5) | 930 (59.6) | 163 (55.3) | 881 (60.8) | 210 (55) |
| Yes | 606 (39.9) | 265 (43.4) | 643 (39.8) | 120 (49.8) | 585 (39.4) | 154 (44.5) | 631 (40.4) | 132 (44.7) | 567 (39.2) | 172 (45) |
|  |  |  |  |  |  |  |  |  |  |  |
|  | Mean (SD) | Mean (SD) | Mean (SD) | Mean (SD) | Mean (SD) | Mean (SD) | Mean (SD) | Mean (SD) | Mean (SD) | Mean (SD) |
| Age | p=0.733 | | p=0.215 | | p=0.215 | | p<0.001 | | p<0.001 | |
|  | 26.1 (6.9) | 26.2 (6.6) | 26.2 (6.9) | 25.6 (6.6) | 26.2 (6.8) | 26.0 (6.9) | 26.4 (6.8) | 24.4 (6.5) | 26.4 (6.9) | 25.2 (6.5) |
| BMI | p=0.121 | | p=0.386 | | p=0.217 | | p=0.251 | | p=0.521 | |
|  | 24.1 (4.6) | 24.4 (4.3) | 24.2 (4.4) | 23.9 (4.3) | 24.1 (4.3) | 24.4 (4.6) | 24.2 (4.4) | 23.9 (4.5) | 24.2 (4.3) | 24.0 (4.7) |

SD= Standard Deviation. P-value for categorical values corresponds to a Chi-squared test for heterogeneity. For continuous data, it corresponds to t-test.
